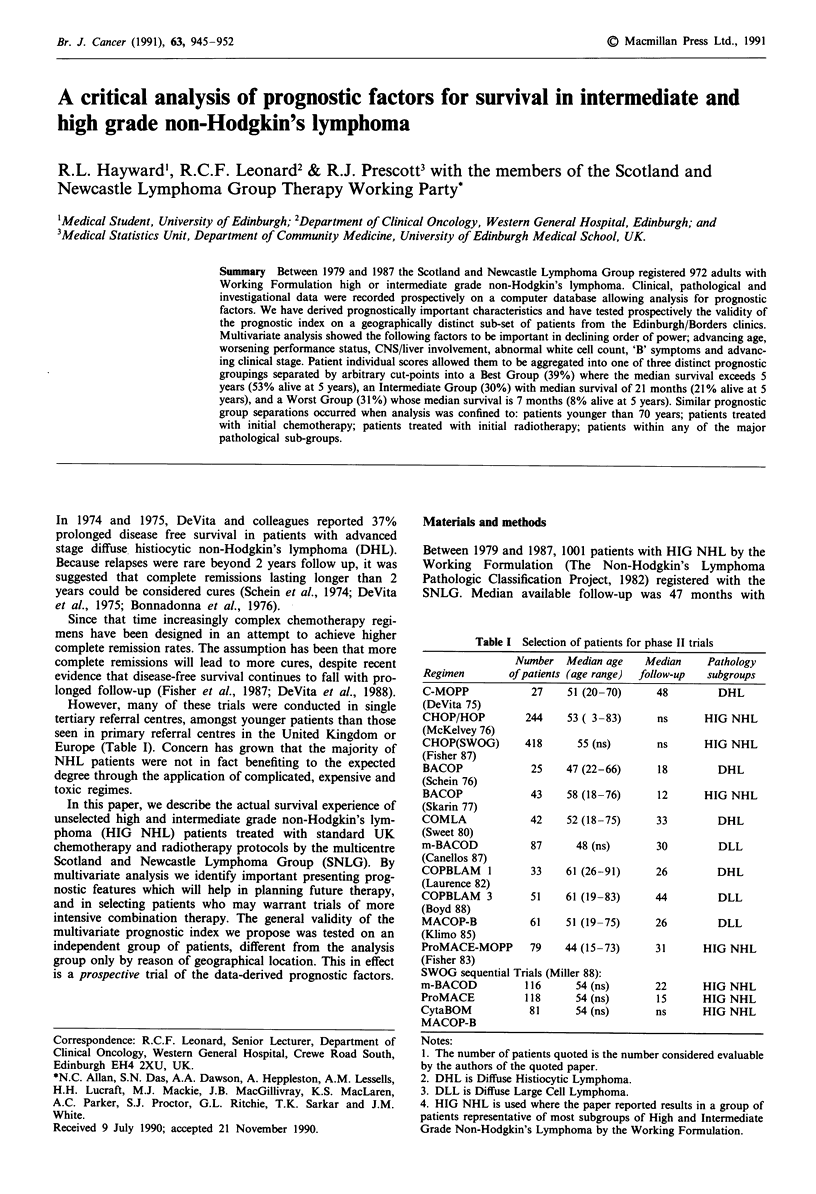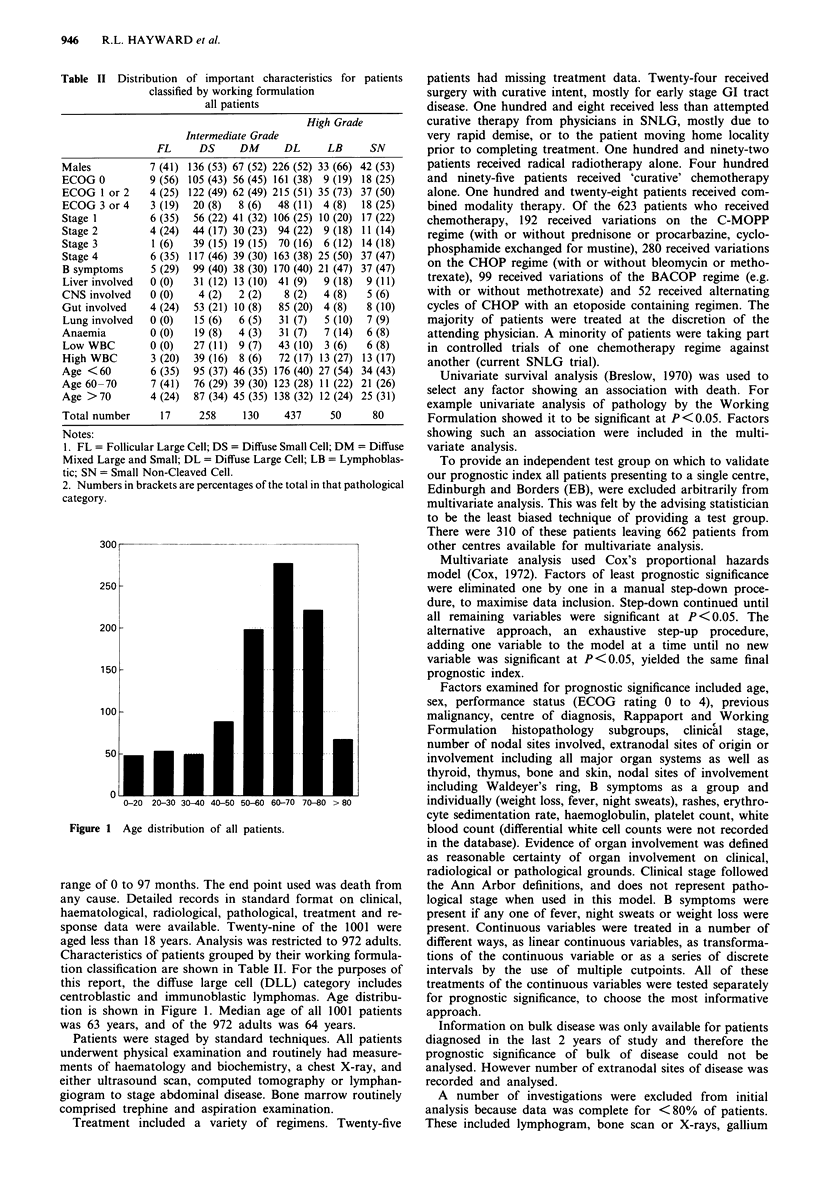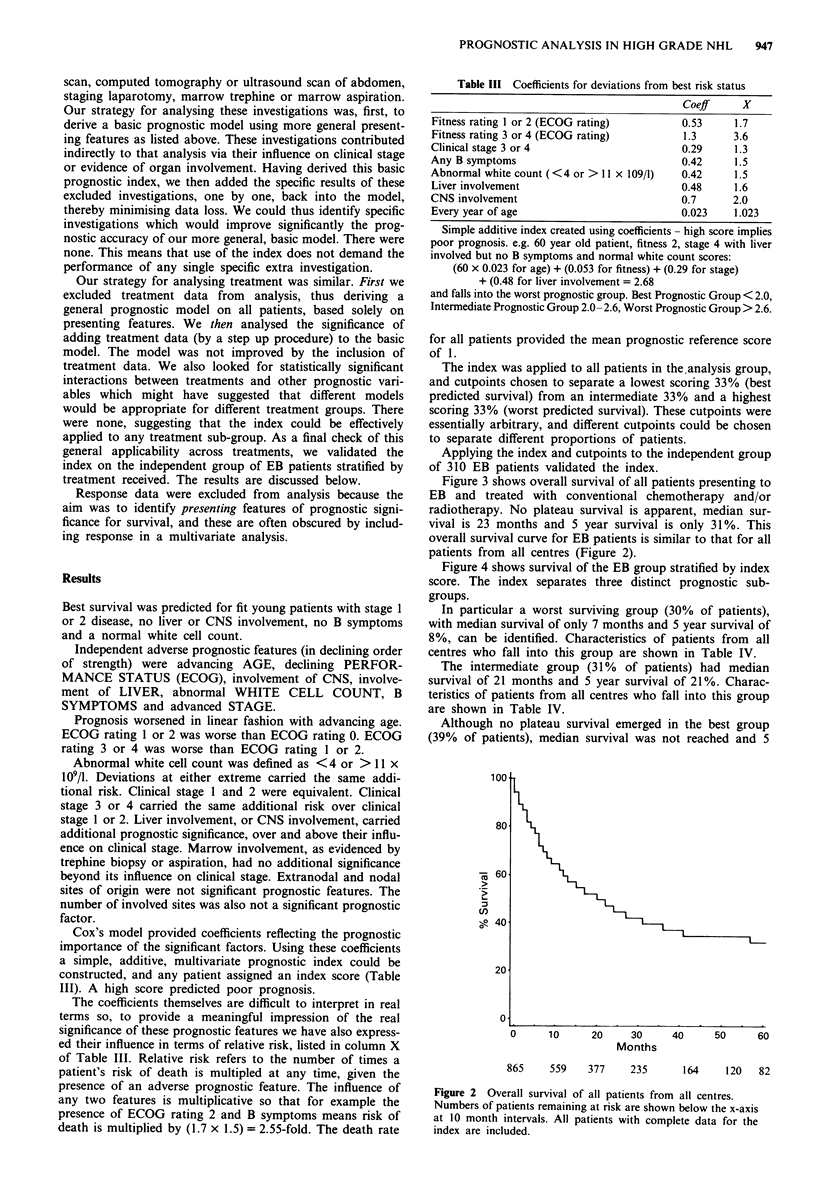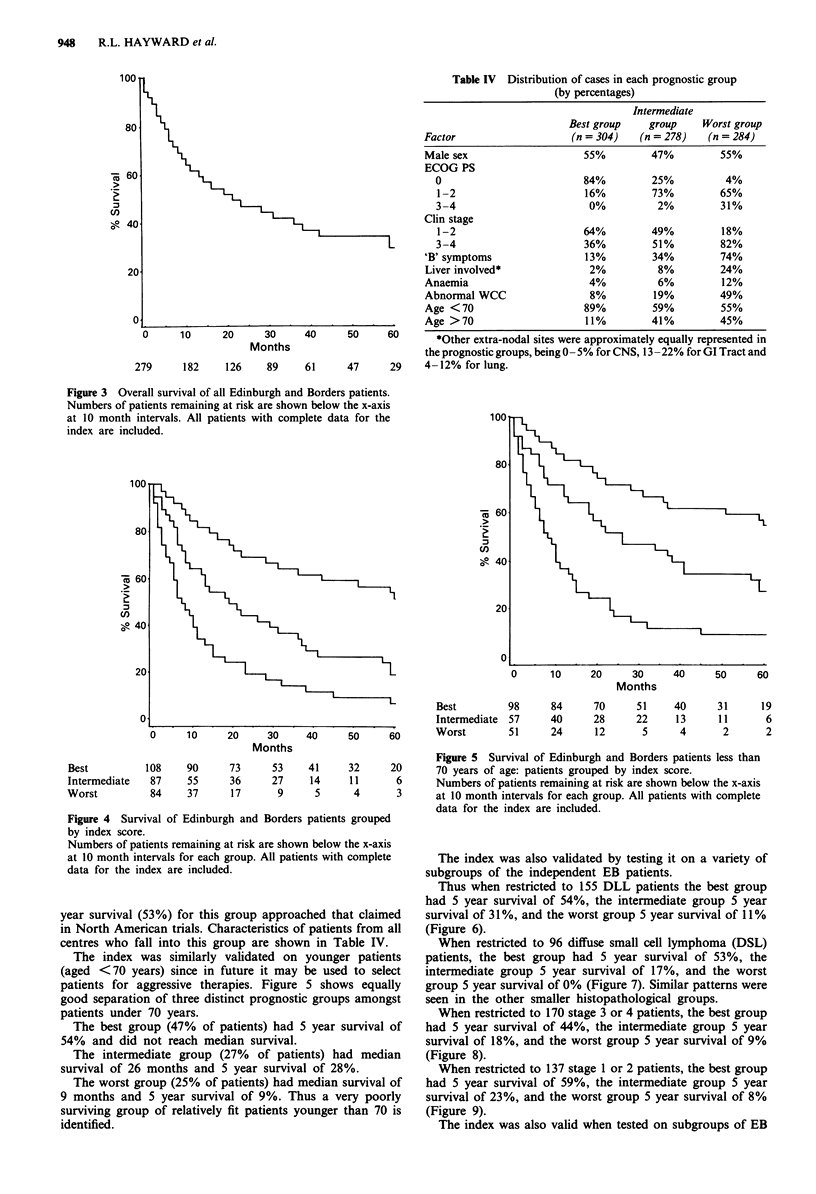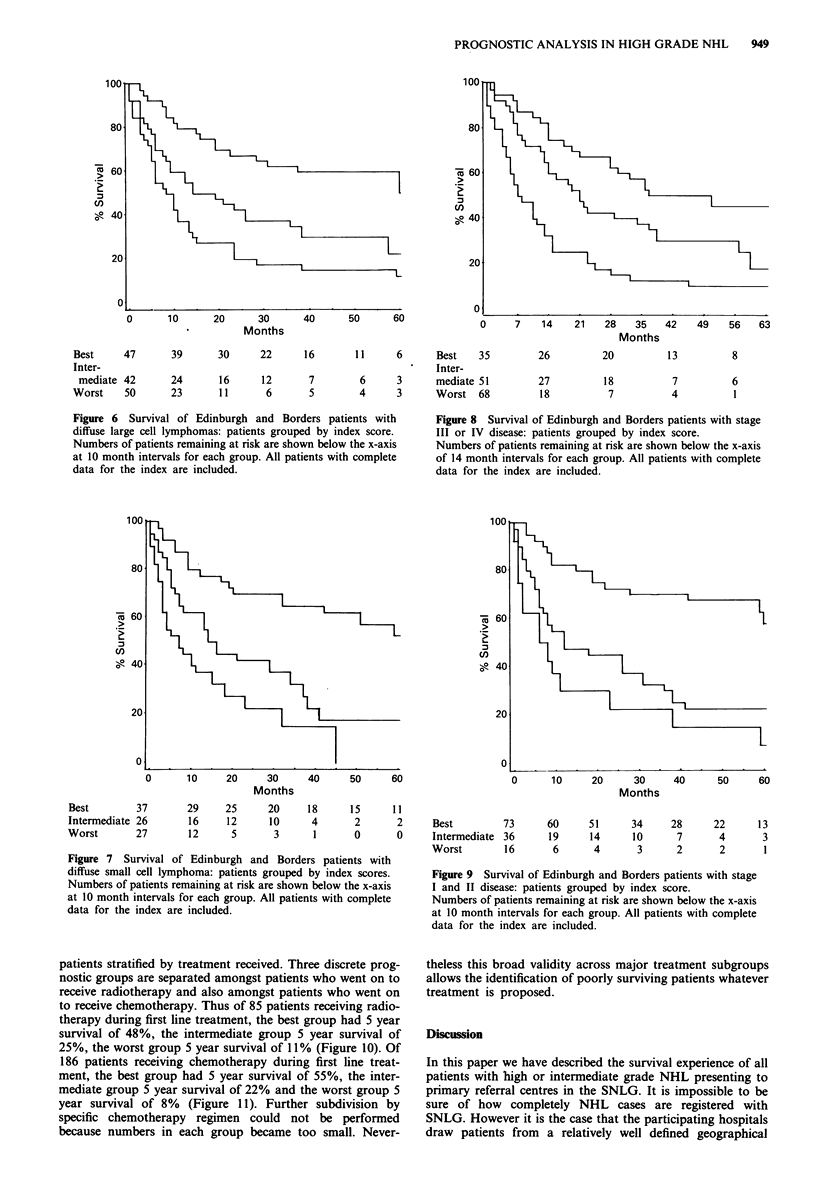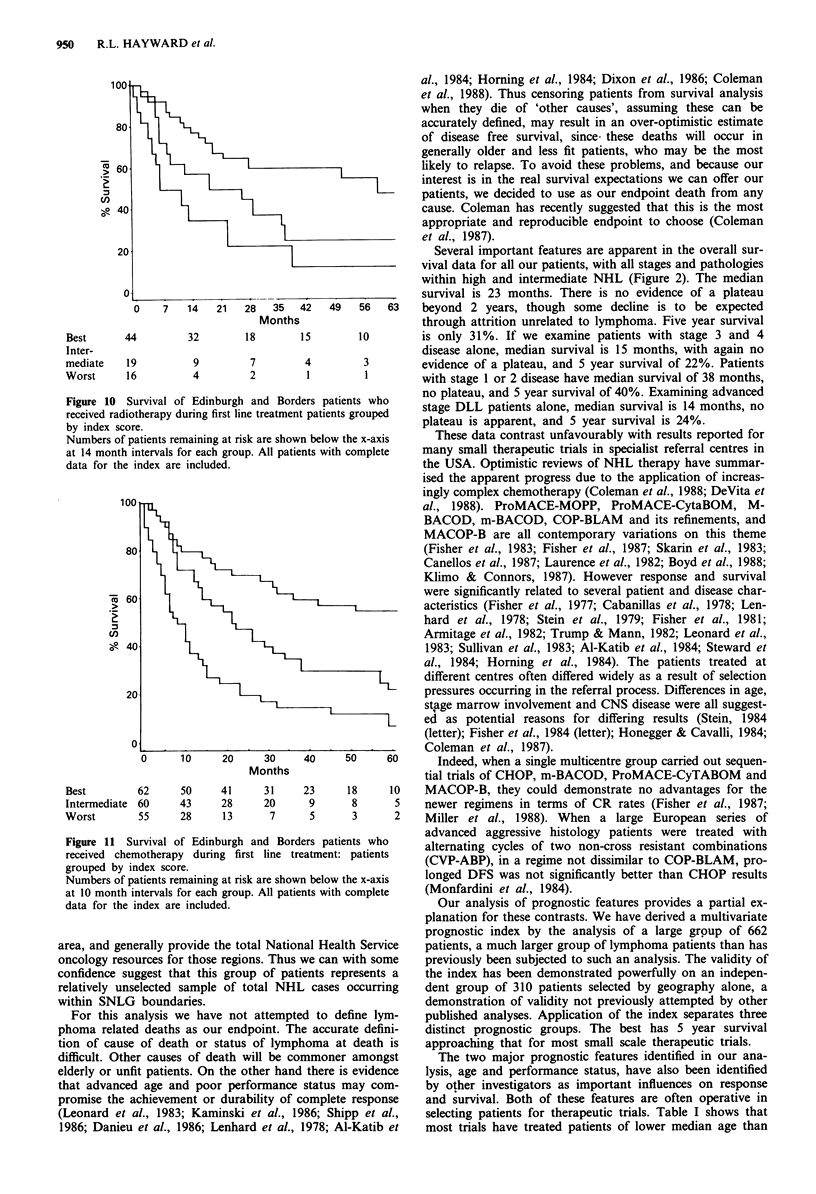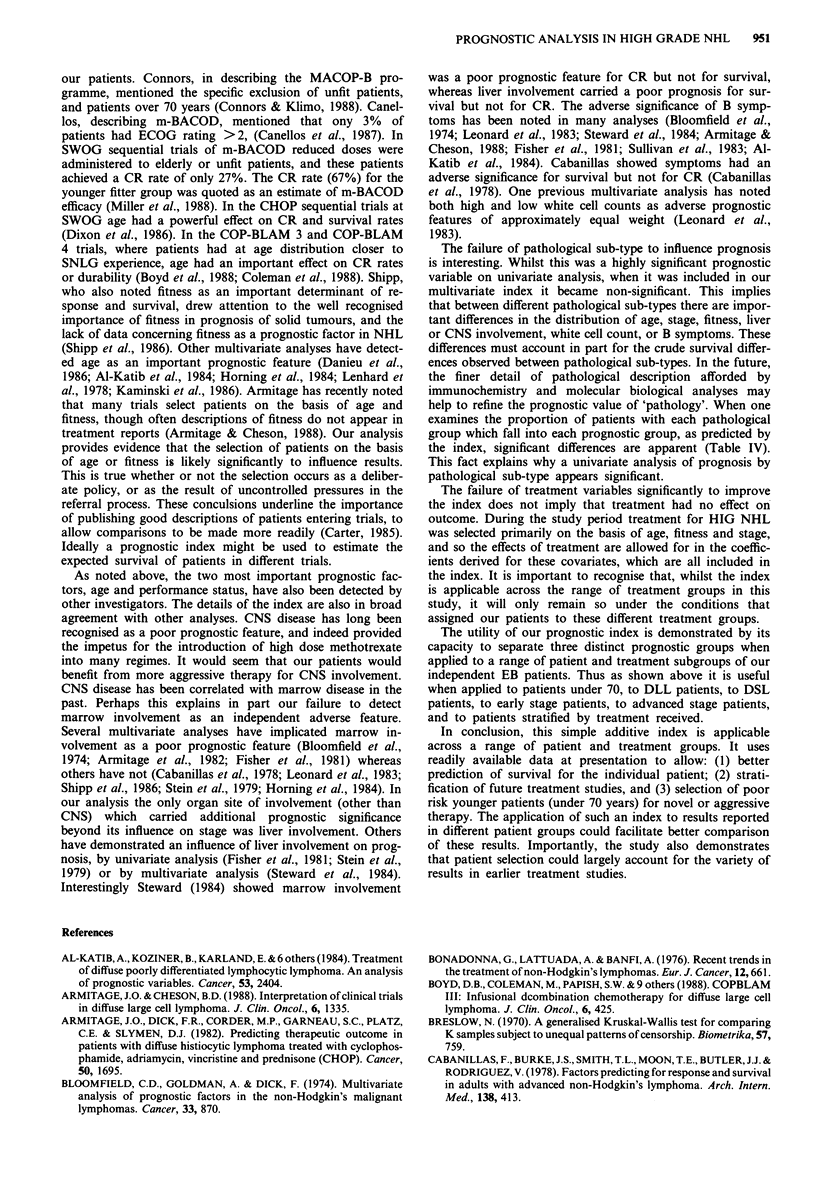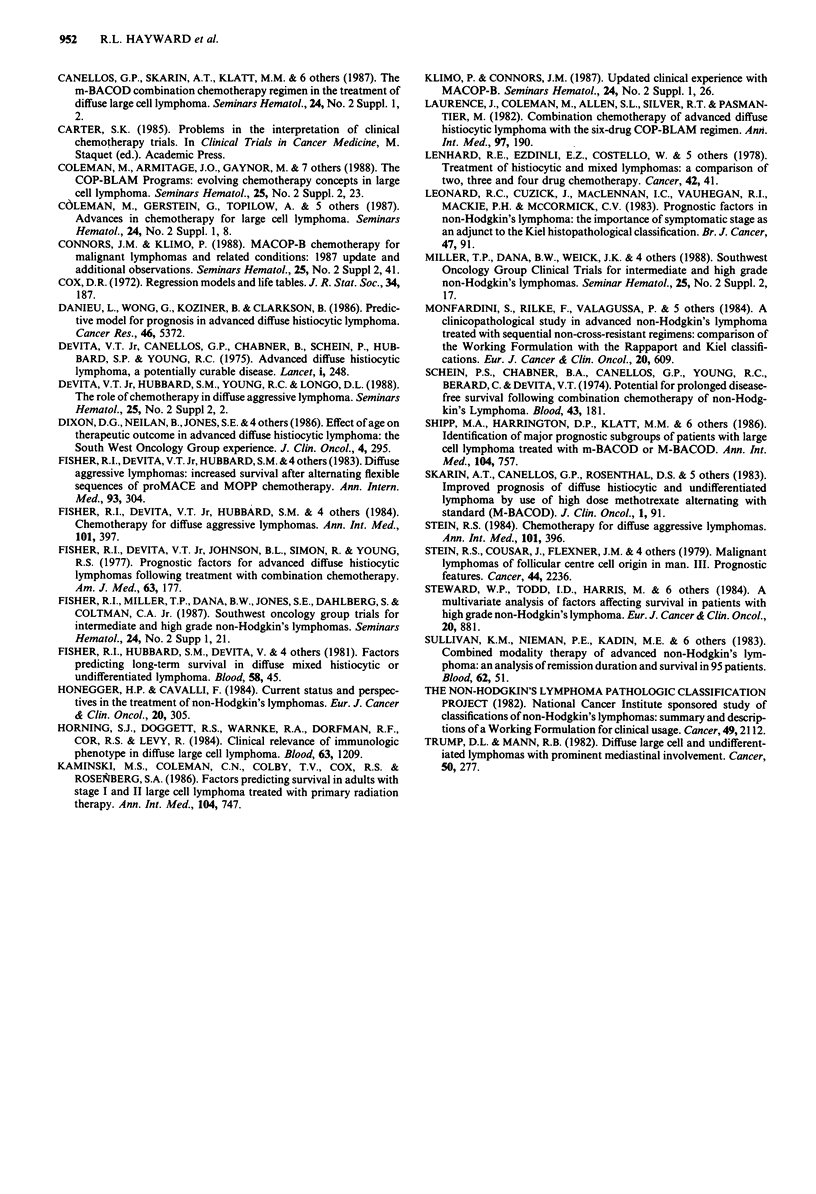# A critical analysis of prognostic factors for survival in intermediate and high grade non-Hodgkin's lymphoma. Scotland and Newcastle Lymphoma Group Therapy Working Party.

**DOI:** 10.1038/bjc.1991.207

**Published:** 1991-06

**Authors:** R. L. Hayward, R. C. Leonard, R. J. Prescott

**Affiliations:** University of Edinburgh, UK.

## Abstract

Between 1979 and 1987 the Scotland and Newcastle Lymphoma Group registered 972 adults with Working Formulation high or intermediate grade non-Hodgkin's lymphoma. Clinical, pathological and investigational data were recorded prospectively on a computer database allowing analysis for prognostic factors. We have derived prognostically important characteristics and have tested prospectively the validity of the prognostic index on a geographically distinct sub-set of patients from the Edinburgh/Borders clinics. Multivariate analysis showed the following factors to be important in declining order of power; advancing age, worsening performance status, CNS/liver involvement, abnormal white cell count, 'B' symptoms and advancing clinical stage. Patient individual scores allowed them to be aggregated into one of three distinct prognostic groupings separated by arbitrary cut-points into a Best Group (39%) where the median survival exceeds 5 years (53% alive at 5 years), an Intermediate Group (30%) with median survival of 21 months (21% alive at 5 years), and a Worst Group (31%) whose median survival is 7 months (8% alive at 5 years). Similar prognostic group separations occurred when analysis was confined to: patients younger than 70 years; patients treated with initial chemotherapy; patients treated with initial radiotherapy; patients within any of the major pathological sub-groups.


Br. J. Cancer (1991), 63, 945–952 © Macmillan Press Ltd., 1991

### A critical analysis of prognostic factors for survival in intermediate and high grade non-Hodgkin's lymphoma

R.L. Hayward[1], R.C.F. Leonard[2] & R.J. Prescott[3] with the members of the Scotland and Newcastle Lymphoma Group Therapy Working Party*

[1]*Medical Student, University of Edinburgh;* [2]*Department of Clinical Oncology, Western General Hospital, Edinburgh; and* [3]*Medical Statistics Unit, Department of Community Medicine, University of Edinburgh Medical School, UK.*

**Summary** Between 1979 and 1987 the Scotland and Newcastle Lymphoma Group registered 972 adults with Working Formulation high or intermediate grade non-Hodgkin's lymphoma. Clinical, pathological and investigational data were recorded prospectively on a computer database allowing analysis for prognostic factors. We have derived prognostically important characteristics and have tested prospectively the validity of the prognostic index on a geographically distinct sub-set of patients from the Edinburgh/Borders clinics. Multivariate analysis showed the following factors to be important in declining order of power; advancing age, worsening performance status, CNS/liver involvement, abnormal white cell count, 'B' symptoms and advancing clinical stage. Patient individual scores allowed them to be aggregated into one of three distinct prognostic groupings separated by arbitrary cut-points into a Best Group (39%) where the median survival exceeds 5 years (53% alive at 5 years), an Intermediate Group (30%) with median survival of 21 months (21% alive at 5 years), and a Worst Group (31%) whose median survival is 7 months (8% alive at 5 years). Similar prognostic group separations occurred when analysis was confined to: patients younger than 70 years; patients treated with initial chemotherapy; patients treated with initial radiotherapy; patients within any of the major pathological sub-groups.

In 1974 and 1975, DeVita and colleagues reported 37% prolonged disease free survival in patients with advanced stage diffuse histiocytic non-Hodgkin's lymphoma (DHL). Because relapses were rare beyond 2 years follow up, it was suggested that complete remissions lasting longer than 2 years could be considered cures (Schein *et al.*, 1974; DeVita *et al.*, 1975; Bonnadonna *et al.*, 1976).

Since that time increasingly complex chemotherapy regimens have been designed in an attempt to achieve higher complete remission rates. The assumption has been that more complete remissions will lead to more cures, despite recent evidence that disease-free survival continues to fall with prolonged follow-up (Fisher *et al.*, 1987; DeVita *et al.*, 1988).

However, many of these trials were conducted in single tertiary referral centres, amongst younger patients than those seen in primary referral centres in the United Kingdom or Europe (Table I). Concern has grown that the majority of NHL patients were not in fact benefiting to the expected degree through the application of complicated, expensive and toxic regimes.

In this paper, we describe the actual survival experience of unselected high and intermediate grade non-Hodgkin's lymphoma (HIG NHL) patients treated with standard UK chemotherapy and radiotherapy protocols by the multicentre Scotland and Newcastle Lymphoma Group (SNLG). By multivariate analysis we identify important presenting prognostic features which will help in planning future therapy, and in selecting patients who may warrant trials of more intensive combination therapy. The general validity of the multivariate prognostic index we propose was tested on an independent group of patients, different from the analysis group only by reason of geographical location. This in effect is a *prospective* trial of the data-derived prognostic factors.

Correspondence: R.C.F. Leonard, Senior Lecturer, Department of Clinical Oncology, Western General Hospital, Crewe Road South, Edinburgh EH4 2XU, UK.

*N.C. Allan, S.N. Das, A.A. Dawson, A. Heppleston, A.M. Lessells, H.H. Lucraft, M.J. Mackie, J.B. MacGillivray, K.S. MacLaren, A.C. Parker, S.J. Proctor, G.L. Ritchie, T.K. Sarkar and J.M. White.

Received 9 July 1990; accepted 21 November 1990.

### Materials and methods

Between 1979 and 1987, 1001 patients with HIG NHL by the Working Formulation (The Non-Hodgkin's Lymphoma Pathologic Classification Project, 1982) registered with the SNLG. Median available follow-up was 47 months with

**Table I** Selection of patients for phase II trials

| *Regimen* | *Number of patients* | *Median age (age range)* | *Median follow-up* | *Pathology subgroups* |
|---|---|---|---|---|
| C-MOPP (DeVita 75) | 27 | 51 (20–70) | 48 | DHL |
| CHOP/HOP (McKelvey 76) | 244 | 53 ( 3–83) | ns | HIG NHL |
| CHOP(SWOG) (Fisher 87) | 418 | 55 (ns) | ns | HIG NHL |
| BACOP (Schein 76) | 25 | 47 (22–66) | 18 | DHL |
| BACOP (Skarin 77) | 43 | 58 (18–76) | 12 | HIG NHL |
| COMLA (Sweet 80) | 42 | 52 (18–75) | 33 | DHL |
| m-BACOD (Canellos 87) | 87 | 48 (ns) | 30 | DLL |
| COPBLAM 1 (Laurence 82) | 33 | 61 (26–91) | 26 | DHL |
| COPBLAM 3 (Boyd 88) | 51 | 61 (19–83) | 44 | DLL |
| MACOP-B (Klimo 85) | 61 | 51 (19–75) | 26 | DLL |
| ProMACE-MOPP (Fisher 83) | 79 | 44 (15–73) | 31 | HIG NHL |
| SWOG sequential Trials (Miller 88): | | | | |
| m-BACOD | 116 | 54 (ns) | 22 | HIG NHL |
| ProMACE | 118 | 54 (ns) | 15 | HIG NHL |
| CytaBOM MACOP-B | 81 | 54 (ns) | ns | HIG NHL |

Notes:
1. The number of patients quoted is the number considered evaluable by the authors of the quoted paper.
2. DHL is Diffuse Histiocytic Lymphoma.
3. DLL is Diffuse Large Cell Lymphoma.
4. HIG NHL is used where the paper reported results in a group of patients representative of most subgroups of High and Intermediate Grade Non-Hodgkin's Lymphoma by the Working Formulation.

**Table II** Distribution of important characteristics for patients classified by working formulation all patients

| | | | | | High Grade | |
|---|---|---|---|---|---|---|
| | | Intermediate Grade | | | | |
| | FL | DS | DM | DL | LB | SN |
| Males | 7 (41) | 136 (53) | 67 (52) | 226 (52) | 33 (66) | 42 (53) |
| ECOG 0 | 9 (56) | 105 (43) | 56 (45) | 161 (38) | 9 (19) | 18 (25) |
| ECOG 1 or 2 | 4 (25) | 122 (49) | 62 (49) | 215 (51) | 35 (73) | 37 (50) |
| ECOG 3 or 4 | 3 (19) | 20 (8) | 8 (6) | 48 (11) | 4 (8) | 18 (25) |
| Stage 1 | 6 (35) | 56 (22) | 41 (32) | 106 (25) | 10 (20) | 17 (22) |
| Stage 2 | 4 (24) | 44 (17) | 30 (23) | 94 (22) | 9 (18) | 11 (14) |
| Stage 3 | 1 (6) | 39 (15) | 19 (15) | 70 (16) | 6 (12) | 14 (18) |
| Stage 4 | 6 (35) | 117 (46) | 39 (30) | 163 (38) | 25 (50) | 37 (47) |
| B symptoms | 5 (29) | 99 (40) | 38 (30) | 170 (40) | 21 (47) | 37 (47) |
| Liver involved | 0 (0) | 31 (12) | 13 (10) | 41 (9) | 9 (18) | 9 (11) |
| CNS involved | 0 (0) | 4 (2) | 2 (2) | 8 (2) | 4 (8) | 5 (6) |
| Gut involved | 4 (24) | 53 (21) | 10 (8) | 85 (20) | 4 (8) | 8 (10) |
| Lung involved | 0 (0) | 15 (6) | 6 (5) | 31 (7) | 5 (10) | 7 (9) |
| Anaemia | 0 (0) | 19 (8) | 4 (3) | 31 (7) | 7 (14) | 6 (8) |
| Low WBC | 0 (0) | 27 (11) | 9 (7) | 43 (10) | 3 (6) | 6 (8) |
| High WBC | 3 (20) | 39 (16) | 8 (6) | 72 (17) | 13 (27) | 13 (17) |
| Age <60 | 6 (35) | 95 (37) | 46 (35) | 176 (40) | 27 (54) | 34 (43) |
| Age 60–70 | 7 (41) | 76 (29) | 39 (30) | 123 (28) | 11 (22) | 21 (26) |
| Age >70 | 4 (24) | 87 (34) | 45 (35) | 138 (32) | 12 (24) | 25 (31) |
| Total number | 17 | 258 | 130 | 437 | 50 | 80 |

Notes:
1. FL = Follicular Large Cell; DS = Diffuse Small Cell; DM = Diffuse Mixed Large and Small; DL = Diffuse Large Cell; LB = Lymphoblastic; SN = Small Non-Cleaved Cell.
2. Numbers in brackets are percentages of the total in that pathological category.

**Figure 1** Age distribution of all patients.

range of 0 to 97 months. The end point used was death from any cause. Detailed records in standard format on clinical, haematological, radiological, pathological, treatment and response data were available. Twenty-nine of the 1001 were aged less than 18 years. Analysis was restricted to 972 adults. Characteristics of patients grouped by their working formulation classification are shown in Table II. For the purposes of this report, the diffuse large cell (DLL) category includes centroblastic and immunoblastic lymphomas. Age distribution is shown in Figure 1. Median age of all 1001 patients was 63 years, and of the 972 adults was 64 years.

Patients were staged by standard techniques. All patients underwent physical examination and routinely had measurements of haematology and biochemistry, a chest X-ray, and either ultrasound scan, computed tomography or lymphangiogram to stage abdominal disease. Bone marrow routinely comprised trephine and aspiration examination.

Treatment included a variety of regimens. Twenty-five patients had missing treatment data. Twenty-four received surgery with curative intent, mostly for early stage GI tract disease. One hundred and eight received less than attempted curative therapy from physicians in SNLG, mostly due to very rapid demise, or to the patient moving home locality prior to completing treatment. One hundred and ninety-two patients received radical radiotherapy alone. Four hundred and ninety-five patients received 'curative' chemotherapy alone. One hundred and twenty-eight patients received combined modality therapy. Of the 623 patients who received chemotherapy, 192 received variations on the C-MOPP regime (with or without prednisone or procarbazine, cyclophosphamide exchanged for mustine), 280 received variations on the CHOP regime (with or without bleomycin or methotrexate), 99 received variations of the BACOP regime (e.g. with or without methotrexate) and 52 received alternating cycles of CHOP with an etoposide containing regimen. The majority of patients were treated at the discretion of the attending physician. A minority of patients were taking part in controlled trials of one chemotherapy regime against another (current SNLG trial).

Univariate survival analysis (Breslow, 1970) was used to select any factor showing an association with death. For example univariate analysis of pathology by the Working Formulation showed it to be significant at $P<0.05$. Factors showing such an association were included in the multivariate analysis.

To provide an independent test group on which to validate our prognostic index all patients presenting to a single centre, Edinburgh and Borders (EB), were excluded arbitrarily from multivariate analysis. This was felt by the advising statistician to be the least biased technique of providing a test group. There were 310 of these patients leaving 662 patients from other centres available for multivariate analysis.

Multivariate analysis used Cox's proportional hazards model (Cox, 1972). Factors of least prognostic significance were eliminated one by one in a manual step-down procedure, to maximise data inclusion. Step-down continued until all remaining variables were significant at $P<0.05$. The alternative approach, an exhaustive step-up procedure, adding one variable to the model at a time until no new variable was significant at $P<0.05$, yielded the same final prognostic index.

Factors examined for prognostic significance included age, sex, performance status (ECOG rating 0 to 4), previous malignancy, centre of diagnosis, Rappaport and Working Formulation histopathology subgroups, clinical stage, number of nodal sites involved, extranodal sites of origin or involvement including all major organ systems as well as thyroid, thymus, bone and skin, nodal sites of involvement including Waldeyer's ring, B symptoms as a group and individually (weight loss, fever, night sweats), rashes, erythrocyte sedimentation rate, haemoglobulin, platelet count, white blood count (differential white cell counts were not recorded in the database). Evidence of organ involvement was defined as reasonable certainty of organ involvement on clinical, radiological or pathological grounds. Clinical stage followed the Ann Arbor definitions, and does not represent pathological stage when used in this model. B symptoms were present if any one of fever, night sweats or weight loss were present. Continuous variables were treated in a number of different ways, as linear continuous variables, as transformations of the continuous variable or as a series of discrete intervals by the use of multiple cutpoints. All of these treatments of the continuous variables were tested separately for prognostic significance, to choose the most informative approach.

Information on bulk disease was only available for patients diagnosed in the last 2 years of study and therefore the prognostic significance of bulk of disease could not be analysed. However number of extranodal sites of disease was recorded and analysed.

A number of investigations were excluded from initial analysis because data was complete for <80% of patients. These included lymphogram, bone scan or X-rays, gallium scan, computed tomography or ultrasound scan of abdomen, staging laparotomy, marrow trephine or marrow aspiration. Our strategy for analysing these investigations was, first, to derive a basic prognostic model using more general presenting features as listed above. These investigations contributed indirectly to that analysis via their influence on clinical stage or evidence of organ involvement. Having derived this basic prognostic index, we then added the specific results of these excluded investigations, one by one, back into the model, thereby minimising data loss. We could thus identify specific investigations which would improve significantly the prognostic accuracy of our more general, basic model. There were none. This means that use of the index does not demand the performance of any single specific extra investigation.

Our strategy for analysing treatment was similar. *First* we excluded treatment data from analysis, thus deriving a general prognostic model on all patients, based solely on presenting features. We *then* analysed the significance of adding treatment data (by a step up procedure) to the basic model. The model was not improved by the inclusion of treatment data. We also looked for statistically significant interactions between treatments and other prognostic variables which might have suggested that different models would be appropriate for different treatment groups. There were none, suggesting that the index could be effectively applied to any treatment sub-group. As a final check of this general applicability across treatments, we validated the index on the independent group of EB patients stratified by treatment received. The results are discussed below.

Response data were excluded from analysis because the aim was to identify *presenting* features of prognostic significance for survival, and these are often obscured by including response in a multivariate analysis.

### Results

Best survival was predicted for fit young patients with stage 1 or 2 disease, no liver or CNS involvement, no B symptoms and a normal white cell count.

Independent adverse prognostic features (in declining order of strength) were advancing AGE, declining PERFORMANCE STATUS (ECOG), involvement of CNS, involvement of LIVER, abnormal WHITE CELL COUNT, B SYMPTOMS and advanced STAGE.

Prognosis worsened in linear fashion with advancing age. ECOG rating 1 or 2 was worse than ECOG rating 0. ECOG rating 3 or 4 was worse than ECOG rating 1 or 2.

Abnormal white cell count was defined as $<4$ or $>11 \times 10^9/l$. Deviations at either extreme carried the same additional risk. Clinical stage 1 and 2 were equivalent. Clinical stage 3 or 4 carried the same additional risk over clinical stage 1 or 2. Liver involvement, or CNS involvement, carried additional prognostic significance, over and above their influence on clinical stage. Marrow involvement, as evidenced by trephine biopsy or aspiration, had no additional significance beyond its influence on clinical stage. Extranodal and nodal sites of origin were not significant prognostic features. The number of involved sites was also not a significant prognostic factor.

Cox's model provided coefficients reflecting the prognostic importance of the significant factors. Using these coefficients a simple, additive, multivariate prognostic index could be constructed, and any patient assigned an index score (Table III). A high score predicted poor prognosis.

The coefficients themselves are difficult to interpret in real terms so, to provide a meaningful impression of the real significance of these prognostic features we have also expressed their influence in terms of relative risk, listed in column X of Table III. Relative risk refers to the number of times a patient's risk of death is multipled at any time, given the presence of an adverse prognostic feature. The influence of any two features is multiplicative so that for example the presence of ECOG rating 2 and B symptoms means risk of death is multiplied by $(1.7 \times 1.5) = 2.55$-fold. The death rate for all patients provided the mean prognostic reference score of 1.

**Table III** Coefficients for deviations from best risk status

| | *Coeff* | *X* |
|---|---|---|
| Fitness rating 1 or 2 (ECOG rating) | 0.53 | 1.7 |
| Fitness rating 3 or 4 (ECOG rating) | 1.3 | 3.6 |
| Clinical stage 3 or 4 | 0.29 | 1.3 |
| Any B symptoms | 0.42 | 1.5 |
| Abnormal white count ($<4$ or $>11 \times 109/l$) | 0.42 | 1.5 |
| Liver involvement | 0.48 | 1.6 |
| CNS involvement | 0.7 | 2.0 |
| Every year of age | 0.023 | 1.023 |

Simple additive index created using coefficients – high score implies poor prognosis. e.g. 60 year old patient, fitness 2, stage 4 with liver involved but no B symptoms and normal white count scores:

$(60 \times 0.023$ for age) + (0.053 for fitness) + (0.29 for stage) + (0.48 for liver involvement = 2.68

and falls into the worst prognostic group. Best Prognostic Group $<2.0$, Intermediate Prognostic Group 2.0–2.6, Worst Prognostic Group $>2.6$.

The index was applied to all patients in the analysis group, and cutpoints chosen to separate a lowest scoring 33% (best predicted survival) from an intermediate 33% and a highest scoring 33% (worst predicted survival). These cutpoints were essentially arbitrary, and different cutpoints could be chosen to separate different proportions of patients.

Applying the index and cutpoints to the independent group of 310 EB patients validated the index.

Figure 3 shows overall survival of all patients presenting to EB and treated with conventional chemotherapy and/or radiotherapy. No plateau survival is apparent, median survival is 23 months and 5 year survival is only 31%. This overall survival curve for EB patients is similar to that for all patients from all centres (Figure 2).

Figure 4 shows survival of the EB group stratified by index score. The index separates three distinct prognostic sub-groups.

In particular a worst surviving group (30% of patients), with median survival of only 7 months and 5 year survival of 8%, can be identified. Characteristics of patients from all centres who fall into this group are shown in Table IV.

The intermediate group (31% of patients) had median survival of 21 months and 5 year survival of 21%. Characteristics of patients from all centres who fall into this group are shown in Table IV.

Although no plateau survival emerged in the best group (39% of patients), median survival was not reached and 5

**Figure 2** Overall survival of all patients from all centres. Numbers of patients remaining at risk are shown below the x-axis at 10 month intervals. All patients with complete data for the index are included.

279 182 126 89 61 47 29

Figure 3 Overall survival of all Edinburgh and Borders patients. Numbers of patients remaining at risk are shown below the x-axis at 10 month intervals. All patients with complete data for the index are included.

| | | | | | | | |
|---|---|---|---|---|---|---|---|
| Best | 108 | 90 | 73 | 53 | 41 | 32 | 20 |
| Intermediate | 87 | 55 | 36 | 27 | 14 | 11 | 6 |
| Worst | 84 | 37 | 17 | 9 | 5 | 4 | 3 |

Figure 4 Survival of Edinburgh and Borders patients grouped by index score.
Numbers of patients remaining at risk are shown below the x-axis at 10 month intervals for each group. All patients with complete data for the index are included.

Table IV Distribution of cases in each prognostic group (by percentages)

| Factor | Best group (n = 304) | Intermediate group (n = 278) | Worst group (n = 284) |
|---|---|---|---|
| Male sex | 55% | 47% | 55% |
| ECOG PS | | | |
| 0 | 84% | 25% | 4% |
| 1-2 | 16% | 73% | 65% |
| 3-4 | 0% | 2% | 31% |
| Clin stage | | | |
| 1-2 | 64% | 49% | 18% |
| 3-4 | 36% | 51% | 82% |
| 'B' symptoms | 13% | 34% | 74% |
| Liver involved* | 2% | 8% | 24% |
| Anaemia | 4% | 6% | 12% |
| Abnormal WCC | 8% | 19% | 49% |
| Age <70 | 89% | 59% | 55% |
| Age >70 | 11% | 41% | 45% |

*Other extra-nodal sites were approximately equally represented in the prognostic groups, being 0-5% for CNS, 13-22% for GI Tract and 4-12% for lung.

| | | | | | | | |
|---|---|---|---|---|---|---|---|
| Best | 98 | 84 | 70 | 51 | 40 | 31 | 19 |
| Intermediate | 57 | 40 | 28 | 22 | 13 | 11 | 6 |
| Worst | 51 | 24 | 12 | 5 | 4 | 2 | 2 |

Figure 5 Survival of Edinburgh and Borders patients less than 70 years of age: patients grouped by index score.
Numbers of patients remaining at risk are shown below the x-axis at 10 month intervals for each group. All patients with complete data for the index are included.

year survival (53%) for this group approached that claimed in North American trials. Characteristics of patients from all centres who fall into this group are shown in Table IV.

The index was similarly validated on younger patients (aged <70 years) since in future it may be used to select patients for aggressive therapies. Figure 5 shows equally good separation of three distinct prognostic groups amongst patients under 70 years.

The best group (47% of patients) had 5 year survival of 54% and did not reach median survival.

The intermediate group (27% of patients) had median survival of 26 months and 5 year survival of 28%.

The worst group (25% of patients) had median survival of 9 months and 5 year survival of 9%. Thus a very poorly surviving group of relatively fit patients younger than 70 is identified.

The index was also validated by testing it on a variety of subgroups of the independent EB patients.

Thus when restricted to 155 DLL patients the best group had 5 year survival of 54%, the intermediate group 5 year survival of 31%, and the worst group 5 year survival of 11% (Figure 6).

When restricted to 96 diffuse small cell lymphoma (DSL) patients, the best group had 5 year survival of 53%, the intermediate group 5 year survival of 17%, and the worst group 5 year survival of 0% (Figure 7). Similar patterns were seen in the other smaller histopathological groups.

When restricted to 170 stage 3 or 4 patients, the best group had 5 year survival of 44%, the intermediate group 5 year survival of 18%, and the worst group 5 year survival of 9% (Figure 8).

When restricted to 137 stage 1 or 2 patients, the best group had 5 year survival of 59%, the intermediate group 5 year survival of 23%, and the worst group 5 year survival of 8% (Figure 9).

The index was also valid when tested on subgroups of EB

| | | | | | | | |
|---|---|---|---|---|---|---|---|
| Best | 47 | 39 | 30 | 22 | 16 | 11 | 6 |
| Intermediate | 42 | 24 | 16 | 12 | 7 | 6 | 3 |
| Worst | 50 | 23 | 11 | 6 | 5 | 4 | 3 |

**Figure 6** Survival of Edinburgh and Borders patients with diffuse large cell lymphomas: patients grouped by index score. Numbers of patients remaining at risk are shown below the x-axis at 10 month intervals for each group. All patients with complete data for the index are included.

| | | | | | |
|---|---|---|---|---|---|
| Best | 35 | 26 | 20 | 13 | 8 |
| Intermediate | 51 | 27 | 18 | 7 | 6 |
| Worst | 68 | 18 | 7 | 4 | 1 |

**Figure 8** Survival of Edinburgh and Borders patients with stage III or IV disease: patients grouped by index score.
Numbers of patients remaining at risk are shown below the x-axis of 14 month intervals for each group. All patients with complete data for the index are included.

| | | | | | | | |
|---|---|---|---|---|---|---|---|
| Best | 37 | 29 | 25 | 20 | 18 | 15 | 11 |
| Intermediate | 26 | 16 | 12 | 10 | 4 | 2 | 2 |
| Worst | 27 | 12 | 5 | 3 | 1 | 0 | 0 |

**Figure 7** Survival of Edinburgh and Borders patients with diffuse small cell lymphoma: patients grouped by index scores. Numbers of patients remaining at risk are shown below the x-axis at 10 month intervals for each group. All patients with complete data for the index are included.

| | | | | | | | |
|---|---|---|---|---|---|---|---|
| Best | 73 | 60 | 51 | 34 | 28 | 22 | 13 |
| Intermediate | 36 | 19 | 14 | 10 | 7 | 4 | 3 |
| Worst | 16 | 6 | 4 | 3 | 2 | 2 | 1 |

**Figure 9** Survival of Edinburgh and Borders patients with stage I and II disease: patients grouped by index score.
Numbers of patients remaining at risk are shown below the x-axis at 10 month intervals for each group. All patients with complete data for the index are included.

patients stratified by treatment received. Three discrete prognostic groups are separated amongst patients who went on to receive radiotherapy and also amongst patients who went on to receive chemotherapy. Thus of 85 patients receiving radiotherapy during first line treatment, the best group had 5 year survival of 48%, the intermediate group 5 year survival of 25%, the worst group 5 year survival of 11% (Figure 10). Of 186 patients receiving chemotherapy during first line treatment, the best group had 5 year survival of 55%, the intermediate group 5 year survival of 22% and the worst group 5 year survival of 8% (Figure 11). Further subdivision by specific chemotherapy regimen could not be performed because numbers in each group became too small. Nevertheless this broad validity across major treatment subgroups allows the identification of poorly surviving patients whatever treatment is proposed.

### Discussion

In this paper we have described the survival experience of all patients with high or intermediate grade NHL presenting to primary referral centres in the SNLG. It is impossible to be sure of how completely NHL cases are registered with SNLG. However it is the case that the participating hospitals draw patients from a relatively well defined geographical

| | | | | | |
|---|---|---|---|---|---|
| Best | 44 | 32 | 18 | 15 | 10 |
| Inter-mediate | 19 | 9 | 7 | 4 | 3 |
| Worst | 16 | 4 | 2 | 1 | 1 |

**Figure 10** Survival of Edinburgh and Borders patients who received radiotherapy during first line treatment patients grouped by index score.
Numbers of patients remaining at risk are shown below the x-axis at 14 month intervals for each group. All patients with complete data for the index are included.

| | | | | | | | |
|---|---|---|---|---|---|---|---|
| Best | 62 | 50 | 41 | 31 | 23 | 18 | 10 |
| Intermediate | 60 | 43 | 28 | 20 | 9 | 8 | 5 |
| Worst | 55 | 28 | 13 | 7 | 5 | 3 | 2 |

**Figure 11** Survival of Edinburgh and Borders patients who received chemotherapy during first line treatment: patients grouped by index score.
Numbers of patients remaining at risk are shown below the x-axis at 10 month intervals for each group. All patients with complete data for the index are included.

area, and generally provide the total National Health Service oncology resources for those regions. Thus we can with some confidence suggest that this group of patients represents a relatively unselected sample of total NHL cases occurring within SNLG boundaries.

For this analysis we have not attempted to define lymphoma related deaths as our endpoint. The accurate definition of cause of death or status of lymphoma at death is difficult. Other causes of death will be commoner amongst elderly or unfit patients. On the other hand there is evidence that advanced age and poor performance status may compromise the achievement or durability of complete response (Leonard *et al.*, 1983; Kaminski *et al.*, 1986; Shipp *et al.*, 1986; Danieu *et al.*, 1986; Lenhard *et al.*, 1978; Al-Katib *et al.*, 1984; Horning *et al.*, 1984; Dixon *et al.*, 1986; Coleman *et al.*, 1988). Thus censoring patients from survival analysis when they die of 'other causes', assuming these can be accurately defined, may result in an over-optimistic estimate of disease free survival, since these deaths will occur in generally older and less fit patients, who may be the most likely to relapse. To avoid these problems, and because our interest is in the real survival expectations we can offer our patients, we decided to use as our endpoint death from any cause. Coleman has recently suggested that this is the most appropriate and reproducible endpoint to choose (Coleman *et al.*, 1987).

Several important features are apparent in the overall survival data for all our patients, with all stages and pathologies within high and intermediate NHL (Figure 2). The median survival is 23 months. There is no evidence of a plateau beyond 2 years, though some decline is to be expected through attrition unrelated to lymphoma. Five year survival is only 31%. If we examine patients with stage 3 and 4 disease alone, median survival is 15 months, with again no evidence of a plateau, and 5 year survival of 22%. Patients with stage 1 or 2 disease have median survival of 38 months, no plateau, and 5 year survival of 40%. Examining advanced stage DLL patients alone, median survival is 14 months, no plateau is apparent, and 5 year survival is 24%.

These data contrast unfavourably with results reported for many small therapeutic trials in specialist referral centres in the USA. Optimistic reviews of NHL therapy have summarised the apparent progress due to the application of increasingly complex chemotherapy (Coleman *et al.*, 1988; DeVita *et al.*, 1988). ProMACE-MOPP, ProMACE-CytaBOM, M-BACOD, m-BACOD, COP-BLAM and its refinements, and MACOP-B are all contemporary variations on this theme (Fisher *et al.*, 1983; Fisher *et al.*, 1987; Skarin *et al.*, 1983; Canellos *et al.*, 1987; Laurence *et al.*, 1982; Boyd *et al.*, 1988; Klimo & Connors, 1987). However response and survival were significantly related to several patient and disease characteristics (Fisher *et al.*, 1977; Cabanillas *et al.*, 1978; Lenhard *et al.*, 1978; Stein *et al.*, 1979; Fisher *et al.*, 1981; Armitage *et al.*, 1982; Trump & Mann, 1982; Leonard *et al.*, 1983; Sullivan *et al.*, 1983; Al-Katib *et al.*, 1984; Steward *et al.*, 1984; Horning *et al.*, 1984). The patients treated at different centres often differed widely as a result of selection pressures occurring in the referral process. Differences in age, stage marrow involvement and CNS disease were all suggested as potential reasons for differing results (Stein, 1984 (letter); Fisher *et al.*, 1984 (letter); Honegger & Cavalli, 1984; Coleman *et al.*, 1987).

Indeed, when a single multicentre group carried out sequential trials of CHOP, m-BACOD, ProMACE-CyTABOM and MACOP-B, they could demonstrate no advantages for the newer regimens in terms of CR rates (Fisher *et al.*, 1987; Miller *et al.*, 1988). When a large European series of advanced aggressive histology patients were treated with alternating cycles of two non-cross resistant combinations (CVP-ABP), in a regime not dissimilar to COP-BLAM, prolonged DFS was not significantly better than CHOP results (Monfardini *et al.*, 1984).

Our analysis of prognostic features provides a partial explanation for these contrasts. We have derived a multivariate prognostic index by the analysis of a large group of 662 patients, a much larger group of lymphoma patients than has previously been subjected to such an analysis. The validity of the index has been demonstrated powerfully on an independent group of 310 patients selected by geography alone, a demonstration of validity not previously attempted by other published analyses. Application of the index separates three distinct prognostic groups. The best has 5 year survival approaching that for most small scale therapeutic trials.

The two major prognostic features identified in our analysis, age and performance status, have also been identified by other investigators as important influences on response and survival. Both of these features are often operative in selecting patients for therapeutic trials. Table I shows that most trials have treated patients of lower median age than our patients. Connors, in describing the MACOP-B programme, mentioned the specific exclusion of unfit patients, and patients over 70 years (Connors & Klimo, 1988). Canellos, describing m-BACOD, mentioned that ony 3% of patients had ECOG rating >2, (Canellos *et al.*, 1987). In SWOG sequential trials of m-BACOD reduced doses were administered to elderly or unfit patients, and these patients achieved a CR rate of only 27%. The CR rate (67%) for the younger fitter group was quoted as an estimate of m-BACOD efficacy (Miller *et al.*, 1988). In the CHOP sequential trials at SWOG age had a powerful effect on CR and survival rates (Dixon *et al.*, 1986). In the COP-BLAM 3 and COP-BLAM 4 trials, where patients had at age distribution closer to SNLG experience, age had an important effect on CR rates or durability (Boyd *et al.*, 1988; Coleman *et al.*, 1988). Shipp, who also noted fitness as an important determinant of response and survival, drew attention to the well recognised importance of fitness in prognosis of solid tumours, and the lack of data concerning fitness as a prognostic factor in NHL (Shipp *et al.*, 1986). Other multivariate analyses have detected age as an important prognostic feature (Danieu *et al.*, 1986; Al-Katib *et al.*, 1984; Horning *et al.*, 1984; Lenhard *et al.*, 1978; Kaminski *et al.*, 1986). Armitage has recently noted that many trials select patients on the basis of age and fitness, though often descriptions of fitness do not appear in treatment reports (Armitage & Cheson, 1988). Our analysis provides evidence that the selection of patients on the basis of age or fitness is likely significantly to influence results. This is true whether or not the selection occurs as a deliberate policy, or as the result of uncontrolled pressures in the referral process. These conculsions underline the importance of publishing good descriptions of patients entering trials, to allow comparisons to be made more readily (Carter, 1985). Ideally a prognostic index might be used to estimate the expected survival of patients in different trials.

As noted above, the two most important prognostic factors, age and performance status, have also been detected by other investigators. The details of the index are also in broad agreement with other analyses. CNS disease has long been recognised as a poor prognostic feature, and indeed provided the impetus for the introduction of high dose methotrexate into many regimes. It would seem that our patients would benefit from more aggressive therapy for CNS involvement. CNS disease has been correlated with marrow disease in the past. Perhaps this explains in part our failure to detect marrow involvement as an independent adverse feature. Several multivariate analyses have implicated marrow involvement as a poor prognostic feature (Bloomfield *et al.*, 1974; Armitage *et al.*, 1982; Fisher *et al.*, 1981) whereas others have not (Cabanillas *et al.*, 1978; Leonard *et al.*, 1983; Shipp *et al.*, 1986; Stein *et al.*, 1979; Horning *et al.*, 1984). In our analysis the only organ site of involvement (other than CNS) which carried additional prognostic significance beyond its influence on stage was liver involvement. Others have demonstrated an influence of liver involvement on prognosis, by univariate analysis (Fisher *et al.*, 1981; Stein *et al.*, 1979) or by multivariate analysis (Steward *et al.*, 1984). Interestingly Steward (1984) showed marrow involvement was a poor prognostic feature for CR but not for survival, whereas liver involvement carried a poor prognosis for survival but not for CR. The adverse significance of B symptoms has been noted in many analyses (Bloomfield *et al.*, 1974; Leonard *et al.*, 1983; Steward *et al.*, 1984; Armitage & Cheson, 1988; Fisher *et al.*, 1981; Sullivan *et al.*, 1983; Al-Katib *et al.*, 1984). Cabanillas showed symptoms had an adverse significance for survival but not for CR (Cabanillas *et al.*, 1978). One previous multivariate analysis has noted both high and low white cell counts as adverse prognostic features of approximately equal weight (Leonard *et al.*, 1983).

The failure of pathological sub-type to influence prognosis is interesting. Whilst this was a highly significant prognostic variable on univariate analysis, when it was included in our multivariate index it became non-significant. This implies that between different pathological sub-types there are important differences in the distribution of age, stage, fitness, liver or CNS involvement, white cell count, or B symptoms. These differences must account in part for the crude survival differences observed between pathological sub-types. In the future, the finer detail of pathological description afforded by immunochemistry and molecular biological analyses may help to refine the prognostic value of 'pathology'. When one examines the proportion of patients with each pathological group which fall into each prognostic group, as predicted by the index, significant differences are apparent (Table IV). This fact explains why a univariate analysis of prognosis by pathological sub-type appears significant.

The failure of treatment variables significantly to improve the index does not imply that treatment had no effect on outcome. During the study period treatment for HIG NHL was selected primarily on the basis of age, fitness and stage, and so the effects of treatment are allowed for in the coefficients derived for these covariates, which are all included in the index. It is important to recognise that, whilst the index is applicable across the range of treatment groups in this study, it will only remain so under the conditions that assigned our patients to these different treatment groups.

The utility of our prognostic index is demonstrated by its capacity to separate three distinct prognostic groups when applied to a range of patient and treatment subgroups of our independent EB patients. Thus as shown above it is useful when applied to patients under 70, to DLL patients, to DSL patients, to early stage patients, to advanced stage patients, and to patients stratified by treatment received.

In conclusion, this simple additive index is applicable across a range of patient and treatment groups. It uses readily available data at presentation to allow: (1) better prediction of survival for the individual patient; (2) stratification of future treatment studies, and (3) selection of poor risk younger patients (under 70 years) for novel or aggressive therapy. The application of such an index to results reported in different patient groups could facilitate better comparison of these results. Importantly, the study also demonstrates that patient selection could largely account for the variety of results in earlier treatment studies.

### References

AL-KATIB, A., KOZINER, B., KARLAND, E. & 6 others (1984). Treatment of diffuse poorly differentiated lymphocytic lymphoma. An analysis of prognostic variables. *Cancer*, **53**, 2404.

ARMITAGE, J.O. & CHESON, B.D. (1988). Interpretation of clinical trials in diffuse large cell lymphoma. *J. Clin. Oncol.*, **6**, 1335.

ARMITAGE, J.O., DICK, F.R., CORDER, M.P., GARNEAU, S.C., PLATZ, C.E. & SLYMEN, D.J. (1982). Predicting therapeutic outcome in patients with diffuse histiocytic lymphoma treated with cyclophosphamide, adriamycin, vincristine and prednisone (CHOP). *Cancer*, **50**, 1695.

BLOOMFIELD, C.D., GOLDMAN, A. & DICK, F. (1974). Multivariate analysis of prognostic factors in the non-Hodgkin's malignant lymphomas. *Cancer*, **33**, 870.

BONADONNA, G., LATTUADA, A. & BANFI, A. (1976). Recent trends in the treatment of non-Hodgkin's lymphomas. *Eur. J. Cancer*, **12**, 661.

BOYD, D.B., COLEMAN, M., PAPISH, S.W. & 9 others (1988). COPBLAM III: Infusional dcombination chemotherapy for diffuse large cell lymphoma. *J. Clin. Oncol.*, **6**, 425.

BRESLOW, N. (1970). A generalised Kruskal-Wallis test for comparing K samples subject to unequal patterns of censorship. *Biometrika*, **57**, 759.

CABANILLAS, F., BURKE, J.S., SMITH, T.L., MOON, T.E., BUTLER, J.J. & RODRIGUEZ, V. (1978). Factors predicting for response and survival in adults with advanced non-Hodgkin's lymphoma. *Arch. Intern. Med.*, **138**, 413.

CANELLOS, G.P., SKARIN, A.T., KLATT, M.M. & 6 others (1987). The m-BACOD combination chemotherapy regimen in the treatment of diffuse large cell lymphoma. *Seminars Hematol.*, **24**, No. 2 Suppl. 1, 2.

CARTER, S.K. (1985). Problems in the interpretation of clinical chemotherapy trials. In *Clinical Trials in Cancer Medicine*, M. Staquet (ed.). Academic Press.

COLEMAN, M., ARMITAGE, J.O., GAYNOR, M. & 7 others (1988). The COP-BLAM Programs: evolving chemotherapy concepts in large cell lymphoma. *Seminars Hematol.*, **25**, No. 2 Suppl. 2, 23.

COLEMAN, M., GERSTEIN, G., TOPILOW, A. & 5 others (1987). Advances in chemotherapy for large cell lymphoma. *Seminars Hematol.*, **24**, No. 2 Suppl. 1, 8.

CONNORS, J.M. & KLIMO, P. (1988). MACOP-B chemotherapy for malignant lymphomas and related conditions: 1987 update and additional observations. *Seminars Hematol.*, **25**, No. 2 Suppl 2, 41.

COX, D.R. (1972). Regression models and life tables. *J. R. Stat. Soc.*, **34**, 187.

DANIEU, L., WONG, G., KOZINER, B. & CLARKSON, B. (1986). Predictive model for prognosis in advanced diffuse histiocytic lymphoma. *Cancer Res.*, **46**, 5372.

DEVITA, V.T. Jr, CANELLOS, G.P., CHABNER, B., SCHEIN, P., HUBBARD, S.P. & YOUNG, R.C. (1975). Advanced diffuse histiocytic lymphoma, a potentially curable disease. *Lancet*, **i**, 248.

DEVITA, V.T. Jr, HUBBARD, S.M., YOUNG, R.C. & LONGO, D.L. (1988). The role of chemotherapy in diffuse aggressive lymphoma. *Seminars Hematol.*, **25**, No. 2 Suppl 2, 2.

DIXON, D.G., NEILAN, B., JONES, S.E. & 4 others (1986). Effect of age on therapeutic outcome in advanced diffuse histiocytic lymphoma: the South West Oncology Group experience. *J. Clin. Oncol.*, **4**, 295.

FISHER, R.I., DEVITA, V.T. Jr, HUBBARD, S.M. & 4 others (1983). Diffuse aggressive lymphomas: increased survival after alternating flexible sequences of proMACE and MOPP chemotherapy. *Ann. Intern. Med.*, **93**, 304.

FISHER, R.I., DEVITA, V.T. Jr, HUBBARD, S.M. & 4 others (1984). Chemotherapy for diffuse aggressive lymphomas. *Ann. Int. Med.*, **101**, 397.

FISHER, R.I., DEVITA, V.T. Jr, JOHNSON, B.L., SIMON, R. & YOUNG, R.S. (1977). Prognostic factors for advanced diffuse histiocytic lymphomas following treatment with combination chemotherapy. *Am. J. Med.*, **63**, 177.

FISHER, R.I., MILLER, T.P., DANA, B.W., JONES, S.E., DAHLBERG, S. & COLTMAN, C.A. Jr. (1987). Southwest oncology group trials for intermediate and high grade non-Hodgkin's lymphomas. *Seminars Hematol.*, **24**, No. 2 Supp 1, 21.

FISHER, R.I., HUBBARD, S.M., DEVITA, V. & 4 others (1981). Factors predicting long-term survival in diffuse mixed histiocytic or undifferentiated lymphoma. *Blood*, **58**, 45.

HONEGGER, H.P. & CAVALLI, F. (1984). Current status and perspectives in the treatment of non-Hodgkin's lymphomas. *Eur. J. Cancer & Clin. Oncol.*, **20**, 305.

HORNING, S.J., DOGGETT, R.S., WARNKE, R.A., DORFMAN, R.F., COR, R.S. & LEVY, R. (1984). Clinical relevance of immunologic phenotype in diffuse large cell lymphoma. *Blood*, **63**, 1209.

KAMINSKI, M.S., COLEMAN, C.N., COLBY, T.V., COX, R.S. & ROSENBERG, S.A. (1986). Factors predicting survival in adults with stage I and II large cell lymphoma treated with primary radiation therapy. *Ann. Int. Med.*, **104**, 747.

KLIMO, P. & CONNORS, J.M. (1987). Updated clinical experience with MACOP-B. *Seminars Hematol.*, **24**, No. 2 Suppl. 1, 26.

LAURENCE, J., COLEMAN, M., ALLEN, S.L., SILVER, R.T. & PASMANTIER, M. (1982). Combination chemotherapy of advanced diffuse histiocytic lymphoma with the six-drug COP-BLAM regimen. *Ann. Int. Med.*, **97**, 190.

LENHARD, R.E., EZDINLI, E.Z., COSTELLO, W. & 5 others (1978). Treatment of histiocytic and mixed lymphomas: a comparison of two, three and four drug chemotherapy. *Cancer*, **42**, 41.

LEONARD, R.C., CUZICK, J., MACLENNAN, I.C., VAUHEGAN, R.I., MACKIE, P.H. & MCCORMICK, C.V. (1983). Prognostic factors in non-Hodgkin's lymphoma: the importance of symptomatic stage as an adjunct to the Kiel histopathological classification. *Br. J. Cancer*, **47**, 91.

MILLER, T.P., DANA, B.W., WEICK, J.K. & 4 others (1988). Southwest Oncology Group Clinical Trials for intermediate and high grade non-Hodgkin's lymphomas. *Seminar Hematol.*, **25**, No. 2 Suppl. 2, 17.

MONFARDINI, S., RILKE, F., VALAGUSSA, P. & 5 others (1984). A clinicopathological study in advanced non-Hodgkin's lymphoma treated with sequential non-cross-resistant regimens: comparison of the Working Formulation with the Rappaport and Kiel classifications. *Eur. J. Cancer & Clin. Oncol.*, **20**, 609.

SCHEIN, P.S., CHABNER, B.A., CANELLOS, G.P., YOUNG, R.C., BERARD, C. & DEVITA, V.T. (1974). Potential for prolonged disease-free survival following combination chemotherapy of non-Hodgkin's Lymphoma. *Blood*, **43**, 181.

SHIPP, M.A., HARRINGTON, D.P., KLATT, M.M. & 6 others (1986). Identification of major prognostic subgroups of patients with large cell lymphoma treated with m-BACOD or M-BACOD. *Ann. Int. Med.*, **104**, 757.

SKARIN, A.T., CANELLOS, G.P., ROSENTHAL, D.S. & 5 others (1983). Improved prognosis of diffuse histiocytic and undifferentiated lymphoma by use of high dose methotrexate alternating with standard (M-BACOD). *J. Clin. Oncol.*, **1**, 91.

STEIN, R.S. (1984). Chemotherapy for diffuse aggressive lymphomas. *Ann. Int. Med.*, **101**, 396.

STEIN, R.S., COUSAR, J., FLEXNER, J.M. & 4 others (1979). Malignant lymphomas of follicular centre cell origin in man. III. Prognostic features. *Cancer*, **44**, 2236.

STEWARD, W.P., TODD, I.D., HARRIS, M. & 6 others (1984). A multivariate analysis of factors affecting survival in patients with high grade non-Hodgkin's lymphoma. *Eur. J. Cancer & Clin. Oncol.*, **20**, 881.

SULLIVAN, K.M., NIEMAN, P.E., KADIN, M.E. & 6 others (1983). Combined modality therapy of advanced non-Hodgkin's lymphoma: an analysis of remission duration and survival in 95 patients. *Blood*, **62**, 51.

THE NON-HODGKIN'S LYMPHOMA PATHOLOGIC CLASSIFICATION PROJECT (1982). National Cancer Institute sponsored study of classifications of non-Hodgkin's lymphomas: summary and descriptions of a Working Formulation for clinical usage. *Cancer*, **49**, 2112.

TRUMP, D.L. & MANN, R.B. (1982). Diffuse large cell and undifferentiated lymphomas with prominent mediastinal involvement. *Cancer*, **50**, 277.